# Understanding the Prescription Drug Costs among People with ADRD and Comorbidities: Evidence from Nationally Representative Datasets 2003‐2021

**DOI:** 10.1002/alz.088458

**Published:** 2025-01-09

**Authors:** Seyeon Jang, Rozalina G. McCoy, Jie Chen

**Affiliations:** ^1^ University of Maryland School of Public Health, College Park, MD USA; ^2^ The Hospital And Public health interdisciplinary research (HAPPY) Lab, School of Public Health, University of Maryland, College Park, MD USA; ^3^ University of Maryland Institute for Health Computing, Bethesda, MD USA

## Abstract

**Background:**

Escalating drug costs are likely to disproportionately burden individuals with Alzheimer’s disease and related dementias (ADRD) as they often have multiple chronic conditions (MCC). This study aimed to estimate the incremental total and out‐of‐pocket (OOP) drug costs associated with ADRD and MCC across the drug cost distributions.

**Method:**

The study used a nationally representative cross‐sectional dataset from 2003 to 2021 Medical Expenditure Panel Survey. The study sample comprised of 83,667 elderly community dwelling individuals aged 65 and above, among which 3,046 individuals had ADRD diagnosis and 2,710 had both ADRD and MCC. To address the skewed distribution of the drug costs, quantile regression models were used to examine the associations between prescription drug costs and having ADRD/MCC across the drug cost distributions at 25^th^, 50^th^, 75^th^, and 90^th^ percentiles, after adjustment for other sociodemographic factors. MCC was defined as having two or more chronic conditions. Survey weights were applied to all estimates.

**Result:**

ADRD patients and those with ADRD and MCC had significantly higher incremental costs at the upper end of the total and OOP drug cost distributions than at the lower end of the distributions, compared to those who had no ADRD diagnosis. The incremental OOP drug costs of ADRD at the 90^th^ percentile were over 14 times higher than that at the 25^th^ percentile ($624.43, *p*<0.001, 90^th^ percentile; $42.66, *p*<0.001, 25^th^ percentile), while the incremental total drug costs of ADRD at the 90^th^ percentile was about 9 times higher than that of ADRD at the 25^th^ percentile ($3,372.27, *p*<0.001, 90^th^ percentile; $381.31, *p*<0.001, 25^th^ percentile). Similarly, the incremental OOP drug costs among individuals with ADRD and MCC at the 90^th^ percentile were 11 times more than that at the 25^th^ percentile ($756.91 vs. $68.55, respectively), while the incremental cost difference for the total drug costs was 7 times higher among individuals with ADRD and MCC at the 90^th^ percentile than those at the 25^th^ percentile ($3,940.53 vs. $548.80, respectively).

**Conclusion:**

The results of the study highlighted that ADRD patients with and without MCC who are heavy prescription drug users face a disproportionately greater burden in OOP payments.

